# Emodin alleviates LPS-induced inflammatory response in lung injury rat by affecting the function of granulocytes

**DOI:** 10.1186/s12950-020-00252-6

**Published:** 2020-08-08

**Authors:** Hongxia Mei, Ying Tao, Tianhao Zhang, Feng Qi

**Affiliations:** 1 Department of Anesthesiology, Qilu Hospital, Cheeloo College of Medicine, Shandong University, Jinan, 250012 Shandong Province China; 2 Department of Anesthesiology, The Second Affiliated Hospital and Yuying Children’s Hospital of Wenzhou Medical University, Wenzhou, 325000 Zhejiang Province China

**Keywords:** Emodin, Granulocytes, Acute lung injury (ALI), Acute respiratory distress syndrome (ARDS)

## Abstract

**Background:**

Acute lung injury (ALI) and/or acute respiratory distress syndrome (ARDS) are critical life-threatening syndromes characterized by the infiltration of a large number of granulocytes (mainly neutrophils) that lead to an excessive inflammatory response. Emodin (Emo) is a naturally occurring anthraquinone derivative and an active ingredient of Chinese medicine. It is believed to have anti-inflammatory effects. In this study, we examined the impact of Emo on the pulmonary inflammatory response and the granulocytes function in a rat model of lipopolysaccharide (LPS)-induced ALI.

**Results:**

Treatment with Emo protected rat against LPS-induced ALI. Compared to untreated rat, Emo-treated rat exhibited significantly ameliorated lung pathological changes and decreased tumor necrosis factor-α (TNF-α) and interleukin-1β (IL-1β). However, Emo has no protective effect on the rat model of acute lung injury with granulocyte deficiency. In addition, treatment with Emo enhanced the bactericidal capacity of LPS-induced granulocytes via the up-regulation of the ability of granulocytes to phagocytize bacteria and generate neutrophil extracellular traps (NETs). Emo also downregulated the respiratory burst and the expression of reactive oxygen species (ROS) in LPS-stimulated granulocytes, alleviating the damage of granulocytes to surrounding tissues. Finally, Emo can accelerate the resolution of inflammation by promoting apoptosis of granulocytes.

**Conclusion:**

Our results provide the evidence that Emo could ameliorates LPS-induced ALI via its anti-inflammatory action by modulating the function of granulocytes. Emo may be a promising preventive and therapeutic agent in the treatment of ALI.

## Background

Acute lung injury (ALI) and its more severe form, acute respiratory distress syndrome (ARDS), are devastating clinical syndromes. Bacterial or viral pneumonia and sepsis are the most common causes of ALI and ARDS, wherein Gram-negative bacteria are a prominent cause [1]. Lipopolysaccharide (LPS), an important component of the outer membrane of Gram-negative bacteria, is one of the main pro-inflammatory reaction factors in ALI and leads to neutrophil recruitment and pulmonary edema [2, 3].

Granulocytes are the most abundant white blood cells, including neutrophils, eosinophils and basophils, most of which are neutrophils. They are essential for killing bacteria and other microorganisms, and they also have a significant role in regulating the inflammatory response. In order to exert its bactericidal function, circulating granulocytes first follow the chemokine gradient to the inflammatory site. They kill the pathogenic microorganisms through mechanisms, such as phagocytosis, secretion of germicidal particles and neutrophil extracellular traps (NETs).

Granulocyte migration through the activated venous wall is a prerequisite for granulocytes entry to interstitial infection injury and emergency sites [4]. The local hydrolysis of JAM-C proteins on vascular endothelial cells by elastase is a necessary factor for granulocytes to pass through the vein wall [5]. One of the hallmarks associated with the antimicrobial and inflammatory actions of granulocytes is the activation of a powerful respiratory burst, during which granulocytes produce and release large quantities of antibacterial peptides, proteases, and reactive oxygen species (ROS) to kill pathogens entrapped inside the vacuole. The phagocyte NADPH oxidase activity and ROS production play a key role in host defense against microbial pathogens [6].

In response to specific stimuli, neutrophils, the main components of granulocytes, extrude modified chromatin structures decorated with specific cytoplasmic and granular proteins called NETs [7]. NETs is a special form of bactericidal method for neutrophils [8]. The engulfment of apoptotic cells by phagocytes is essential for maintaining normal tissue homeostasis and a prerequisite for the resolution of inflammation [9]. The removal of apoptotic granulocytes from circulation regulates granulopoiesis, and prevents secondary lysis and spillage of noxious granulocyte substances [10, 11].

Rhubarb is one of the most commonly used herbs in traditional Chinese medicine. Emodin (Emo) is mainly extracted from rhizome and root of rhubarb. Its chemical name is 1,3,8-trihydroxy-6-methyl-anthraquinone, and its molecular weight is 270.23. Its chemical structure belongs to the hydroxyindole family. In recent years, studies have found that Emo has a wide range of pharmacological effects, mainly anti-tumor, anti-microbial, anti-oxidation, and anti-inflammatory [12–15]. Modern researches show that Emo extract could significantly inhibit the inflammatory responses in a variety of inflammatory animal models [16]. This is achieved by directly or indirectly inhibiting the activity of inflammatory cells. Currently, the study of Emo on inflammatory cells is mainly focused on macrophages. Its modulation effects on other immune cells, such as granulocytes which play a very important role in lung inflammation-related disease, are not fully elucidated.

This study was designed to investigate whether Emo could exert protective effects on LPS-induced ALI in vivo and whether Emo plays a protective role by affecting the function of granulocytes. In addition, we investigated the effect of different doses of Emo on the function of granulocytes in vitro.

## Results

### Effects of Emo on the proliferation of rat granulocytes

**Fig. 1:**
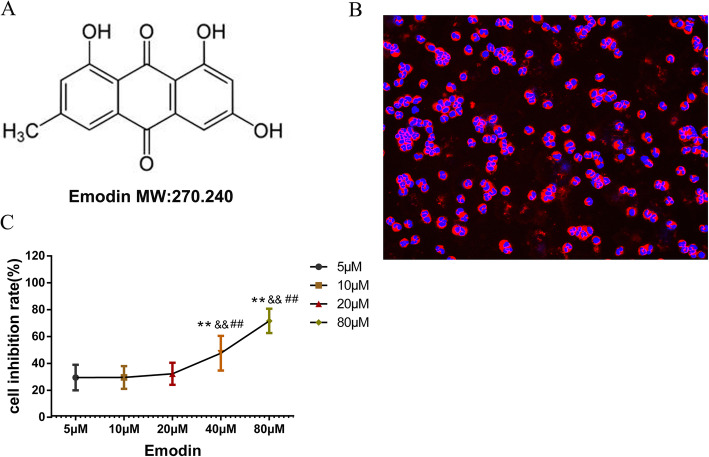
**a** Schematic diagram of Emo; **b** Immunofluorescence staining of rat granulocytes. (C) CCK8 assay. The effect of different concentrations of Emo on granulocytes Cytotoxicity in rats was examined. The data are presented as the mean ± SD. *n* = 6. **P < 0.01 versus Emo 5 μM group; ^&&^*P* < 0.01 versus Emo 10 μM group; ^##^*P* < 0.01 versus Emo 20 μM group

As shown in Fig. 1a, the structure of Emo shows that it belongs to the terpenoid family. Pure rat granulocytes can be obtained by gradient centrifugation as mentioned above. Figure 1b shows rat granulocytes after immunofluorescence staining. Red is granulocyte MPO and blue is granulocyte lobulated nucleus. The ratio of rat granulocytes was quantified, and the purity could reach 94.1% of the isolated cells. Subsequently, different concentrations of Emo were administrated to the granulocytes for 4 h. As shown in Fig. 1c, Emo at the concentration of 40 μ M and 80 μ M had high cytotoxic effect on granulocytes, while the lower concentration of Emo had little cytotoxic effect on granulocytes. EMO of 5 μ M, 10 μ M and 20 μ M was selected for further study.

### Emo protected lung tissues from LPS-induced ALI

**Fig. 2:**
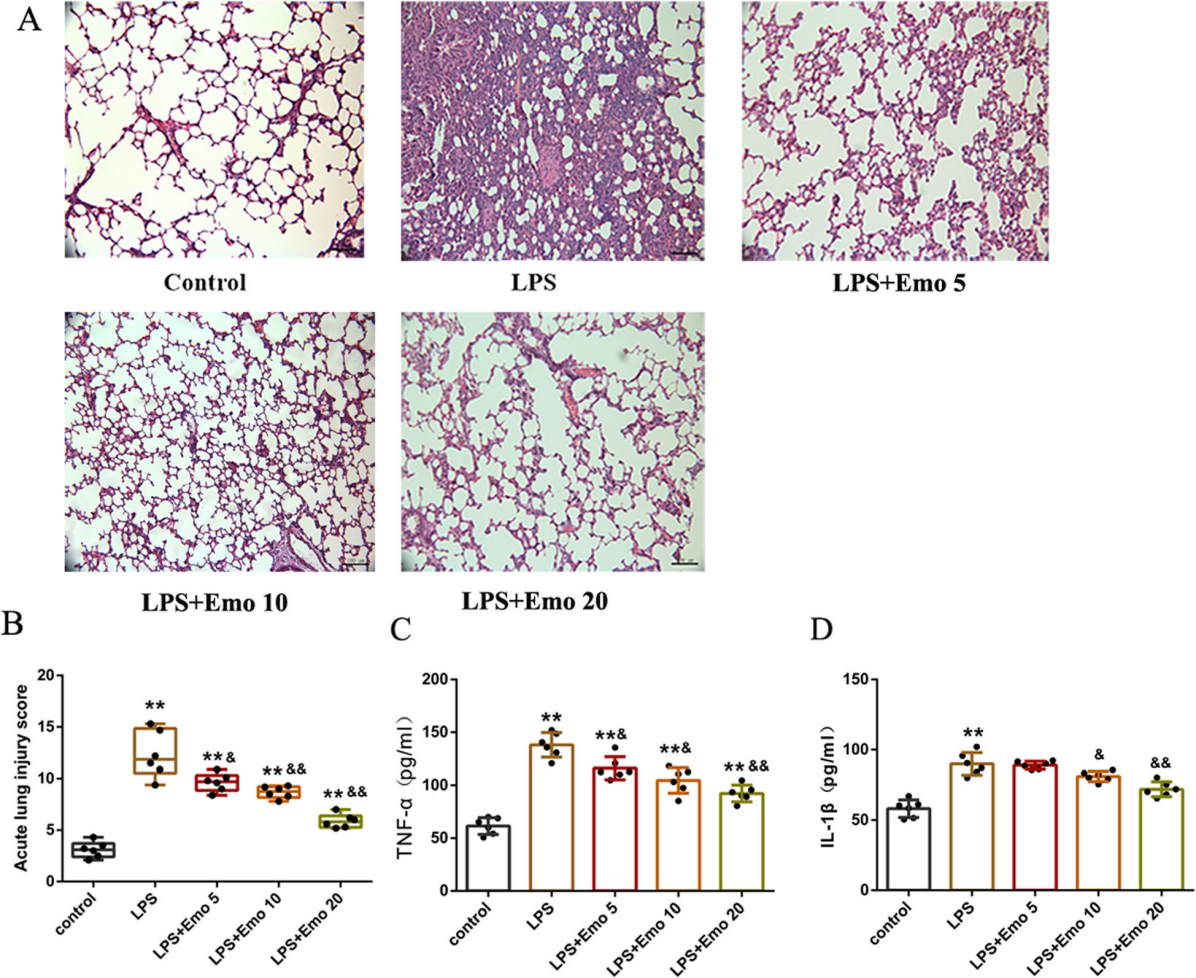
Emo protected lung tissues in LPS-induced ALI. **a** The lung tissues were obtained immediately after exsanguination (4 h after LPS), and the effect of Emo was assessed histologically in H&E-stained sections (original magnification × 200). **b** Lung injury scores were recorded from 0 to 16 according to the criteria described in Materials and Methods. **c** The lung tissue homogenate TNF-α protein expression and **d** the lung tissue homogenate IL-1βprotein expression. The data are presented as the mean ± SD. n = 6. **P < 0.01 versus control group; ^&^*P* < 0.01 versus LPS group; ^&&^*P* < 0.01 versus LPS group

First, we evaluated the effect of Emo on LPS-induced ALI. The control group revealed normal pulmonary histology (Fig. 2a). In contrast, lung tissues in the LPS group were significantly damaged, with interstitial edema, hemorrhaging, thickening of the alveolar wall, and infiltration of inflammatory cells into the interstitium and alveolar spaces, as evidenced by an increase in lung injury score (*p* < 0.01). Compared with the control group, all the morphologic changes observed were less pronounced in the LPS + Emo 5 mg/kg group, LPS + Emo 10 mg/kg group and LPS + Emo 20 mg/kg group. Emo 5 mg/kg attenuated LPS-induced pathologic changes as shown by the decrease in lung injury score (*p* < 0.05). As Emo dosage was increased, the attenuation of LPS-induced pathologic also increased. 10 mg/kg and 20 mg/kg Emo could significantly attenuate LPS-induced pathologic changes(*p* < 0.01) (Fig. 2b).

As expected, the concentrations of TNF-α and IL-1β in the lung tissue homogenate were significantly higher in the LPS group than in the control group. By comparison, the level of TNF in LPS + Emo 5 mg/kg group, LPS + Emo 10 mg/kg group and LPS + Emo 20 mg/kg group decreased in varying degrees, and the decrease of TNF level in LPS + Emo 20 mg/kg group was the most significant (Fig. 2c). After treatment with Emo 10 mg/kg and Emo 20 mg/kg, the level of IL-1 in lung homogenate was also decreased, and Emo 20 mg/kg could significantly reduce the level of IL-1(Fig. 2d).

### Emo impact on respiratory burst and ROS production of granulocytes

**Fig. 3:**
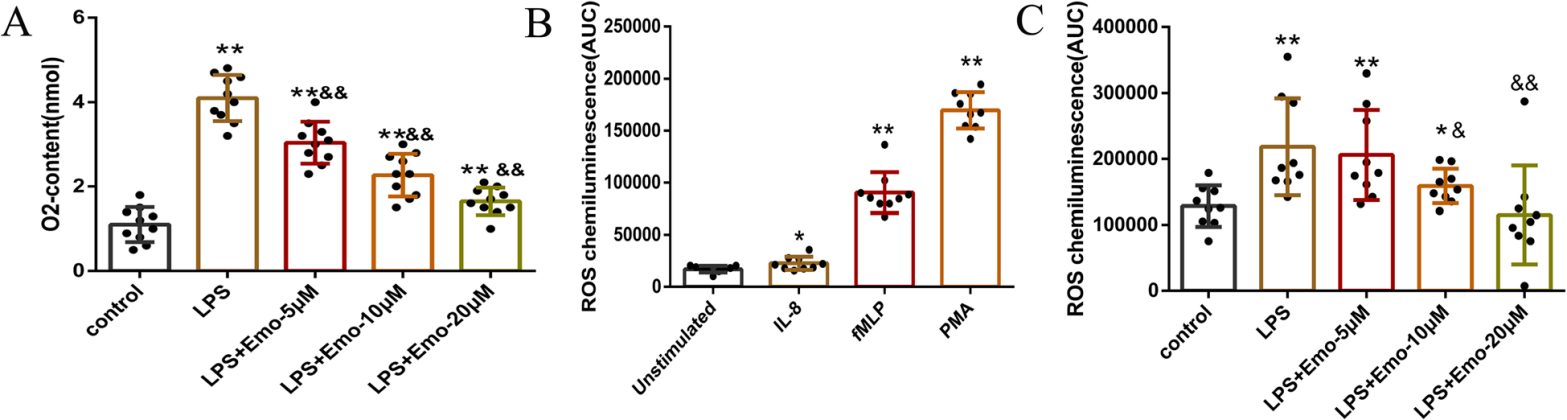
Effect of Emo on respiratory burst and ROS production of granulocytes. **a** Effect of Emo on respiratory burst in rats. **b** Comparison of the ROS level produced by granulocytes after stimulation with IL-8, fMLP, and PMA. **c** Comparison of the ROS level production by granulocytes stimulated by Emo at different concentrations (5 μM, 10 μM and 20 μM). The data are presented as the mean ± SD. *n* = 10. *P < 0.05 versus control group; **P < 0.01 versus control group; ^&^*P* < 0.05 versus LPS group; ^&&^*P* < 0.01 versus LPS group

As shown in Fig. 3a, the level of O_2_^−^ in which the LPS group has a very significant boosting effect on the production of neutrophil O_2_^−^ following fMLP stimulation was detected (*P* < 0.01). Emo decreased the production of O_2_^−^ in a concentration-dependent manner (*P* < 0.01) (Fig. 3a).

The expression of ROS in granulocytes was measured by Luminometer (Fig. 3b-c). According to the literature, we found that there are four groups of agents that can induce ROS production. The first group of priming agents is composed of physiological inflammatory agents, such as C5a, or formylated peptides/proteins such as fMLP. The second group of priming agents is composed of proinflammatory cytokines and adipokines, such as tumor necrosis factor (TNF-α), IL-8. The third group of priming agents is composed of TLR agonists, such as lipopolysaccharide (LPS or endotoxin). The four group of priming agents is Phorbol ester (PMA). Therefore, we chose three agents IL-8、fMLP and PMA to stimulate granulocytes to produce ROS.

It was found that PMA was the best at stimulating granulocytes to produce ROS (*P* < 0.01) (Fig. 3b). Therefore, in the following experiments, PMA was used as an inducer to stimulate granulocytes to produce ROS. Granulocytes were divided into five groups: control group, LPS group (100 ng/ml), LPS + Emo 5 μM group, LPS + Emo 10 μM group and LPS + Emo 20 μM group. Stimulation with Emo was performed for 30 min prior to LPS treatment. PMA (1.5 ng/ml) was used to treat the cells respectively. The ROS production of granulocytes was detected according to the above procedure. In this experiment, the concentration of ROS in the LPS group was significantly increased compared with the control group (*P* < 0.01). Compared with the LPS group, 10 μM Emo and 20 μM Emo reduced ROS production, with 20 μM Emo having an even better effect (*P* < 0.01). At 5 μM, Emo has no effect (*P* > 0.05) (Fig. 3c).

### Emo effect on the release of elastase and NETs production of granulocytes

**Fig. 4:**
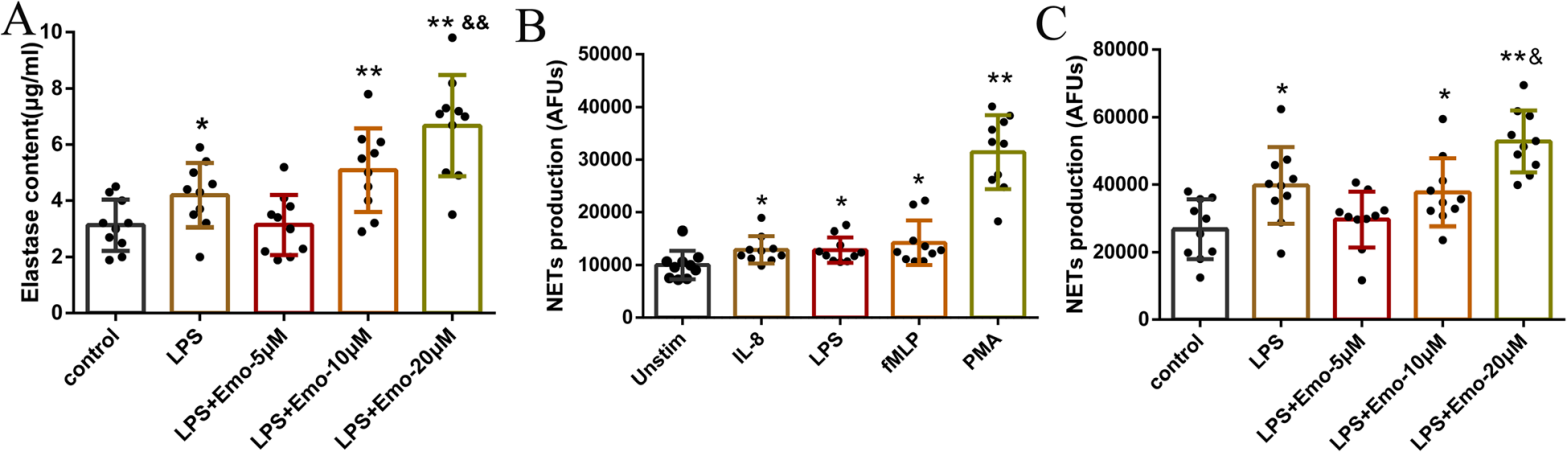
Effect of Emo on the release of elastase and NETs production. **a** The detection of elastase release was mainly carried out by Elastase Activity Assay kit. **b** Comparison of the NETs produced by granulocytes after stimulation with IL-8, LPS, fMLP, and PMA. **c** Comparison of the NETs production by granulocytes stimulated by Emo at different concentrations (5 μM, 10 μM and 20 μM). The data are presented as the mean ± SD. n = 10. *P < 0.05versus control group; **P < 0.01 versus control group; ^&^P<0.05 versus LPS group; ^&&^*P* < 0.01 versus LPS group

As shown in Fig. 4a, the amount of elastase released was significantly higher in the LPS group following stimulation with fMLP than in the control group (*P* < 0.01). This indicates that granulocytes increase the release of elastase in order to fight infection. Elastase release in LPS + Emo 10 μ M and LPS + Emo 20 μ M groups were significantly higher than control group and LPS group (*P* < 0.01).

The literature also confirmed that four group of reagents that induce ROS production can also stimulate the production of NETs, so we also choose four agents LPS, IL-8, fMLP and PMA to induce the production of NETs. Our study found that PMA stimulates granulocytes to produce the most NETs compared with IL-8, LPS and fMLP (*P* < 0.01) (Fig. 4b). As a result, PMA was used as the inducer.

Therefore, in the following experiments, PMA was used as an inducer to stimulate granulocytes to produce NETs. Granulocytes were divided into five groups: control group, LPS group (100 ng/ml), LPS + Emo 5 μM group, LPS + Emo 10 μM group and LPS + Emo 20 μM group. Stimulation with Emo was performed for 30 min prior to LPS treatment. PMA (1.5 ng/ml) was used to treat the cells respectively. The NETs production of granulocytes was detected according to the above procedure. This study found that after PMA stimulation, the generation of NETs in the LPS group increased significantly compared with the control group (*P* < 0.05). In 5 μM Emo and 10 μM Emo groups, the generation of NETs did not increase compared with the LPS group. However, 20 μM Emo significantly increased the production of NETs compared with the LPS group (*P* < 0.05) (Fig. 4c).

### Emo promotes phagocytosis of granulocytes

**Fig. 5:**
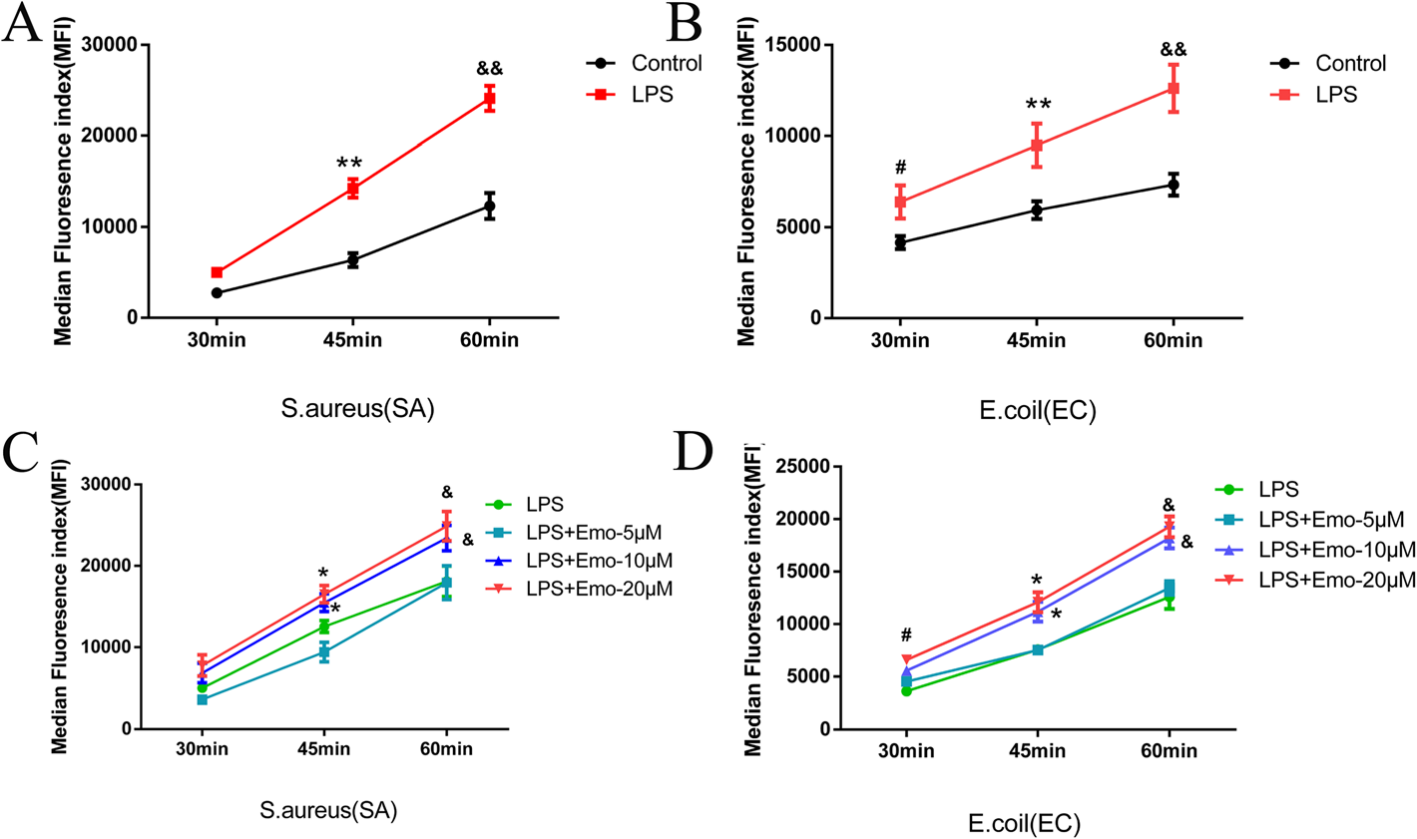
Effect of Emo on phagocytosis of granulocytes. **a** The granulocyte phagocytosis of pHrodo Green S.aureus was tested with flow cytometry. **b** The granulocyte phagocytosis of pHrodo Red E.coli was tested with flow cytometry. The data are presented as the mean ± SD. n = 10. ^#^*P* < 0.05versus LPS group in 30 min; **P < 0.01 versus LPS group in 45 min; ^&&^*P* < 0.01 versus LPS group in 60 min. **c** Comparison of the phagocytosis of pHrodo Green S.aureus by granulocytes stimulated by Emo at different concentrations (5 μM, 10 μM and 20 μM).**d** Comparison of the phagocytosis of pHrodo Red E.coli by neutrophils stimulated by Emo at different concentrations (5 μM, 10 μM and 20 μM). The data are presented as the mean ± SD. n = 10. ^#^*P* < 0.05versus LPS group in 30 min;*P < 0.05versus LPS group in 45 min; ^&^*P* < 0.05 versus LPS group in 60 min

Granulocytes were isolated and adjusted concentration to 1 × 10^6^/ml. After administration of LPS and Emo, granulocytes were co-cultured with pHrodo red E.coli and pHrodo green S.aureus for 30 min, 45 min, and 60 min. We found that the average fluorescence intensity of granulocytes phagocytosis of pHrodo green S.aureus increased from 2749.44 ± 469.95 at 30 min to 12,305.01 ± 1425.02 at 60 min (*P* < 0.01) (Fig. 5a). In addition, the average fluorescence intensity of granulocytes phagocytosis of pHrodo red E.coli increased from 4159.30 ± 357.72 at 30 min to 7340.26 ± 597.80 at 60 min (*P* < 0.01) (Fig. 5b). This result suggests that granulocytes phagocytosis of pHrodo green S.aureus and pHrodo red E.coli increases with time, reaching a peak at 60 min. We also found that LPS-stimulated granulocytes showed increased phagocytosis of pHrodo green S.aureus compared with the control group at 45 min and 60 min (*P* < 0.01) (Fig. 5a). While the phagocytosis of granulocytes to pHrodo red E.coli at 30 min, 45 min and 60 min was higher than control group, especially at 60 min (*P* < 0.05 & *P* < 0.01) (Fig. 5b). In the groups treated with Emo, the experimental results showed that the granulocyte phagocytosis of pHrodo green S.aureus and pHrodo red E.coli at 45 min and 60 min was significantly enhanced for the 10 μM and 20 μM Emo groups (*P* < 0.05) (Fig. 5c-d).

### Emo promotes apoptosis of granulocytes

**Fig. 6:**
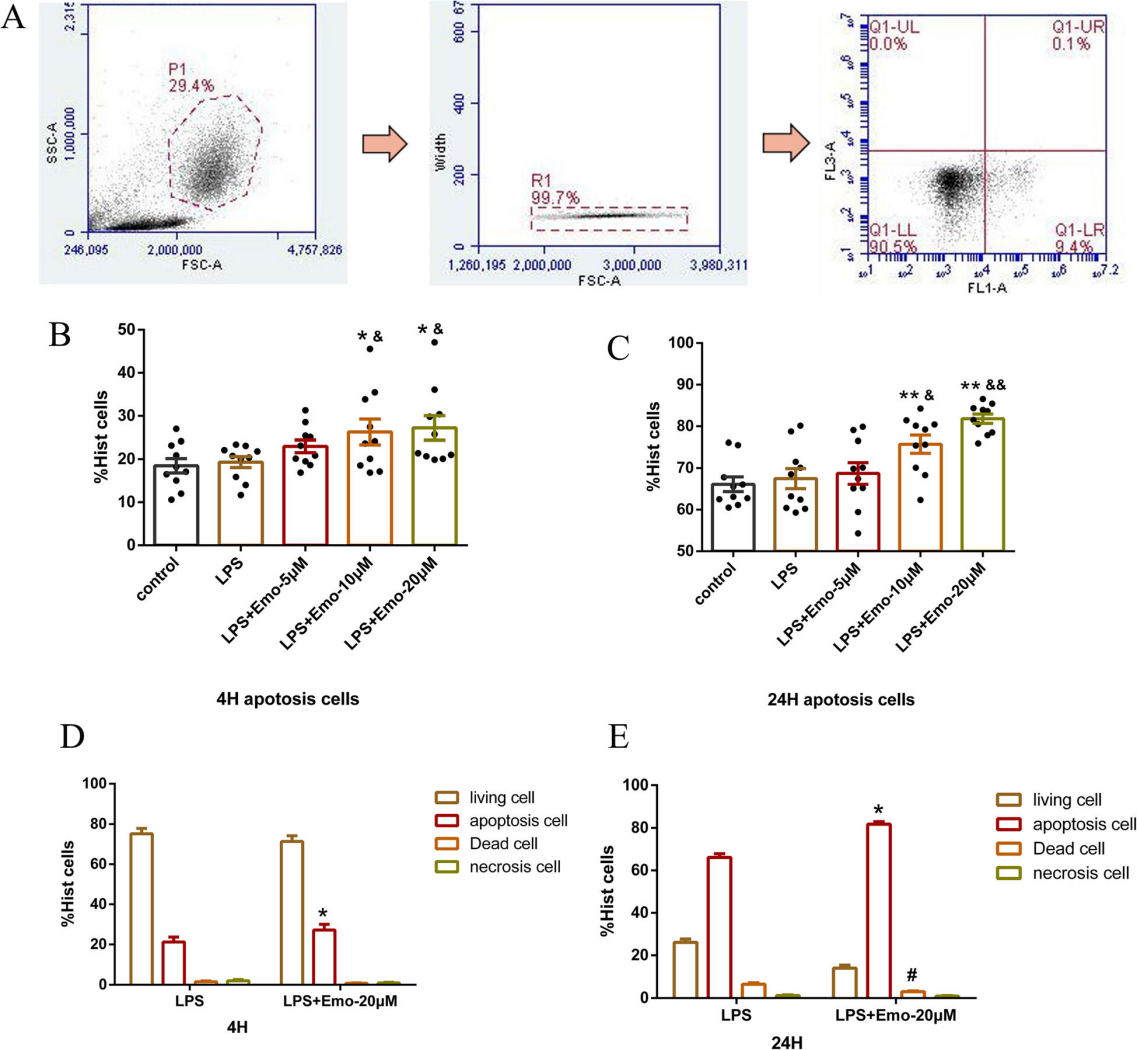
Effect of Emo on apoptosis of granulocytes. **a** The apoptosis of granulocytes was measured by flow cytometry. **b** The proportion of apoptosis of granulocytes in each group at 4 h was measured. **c** The proportion of apoptosis of granulocytes in each group at 24 h was measured. The data are presented as the mean ± SD. n = 10. *P<0.05versus control group; **P < 0.01 versus LPS group; ^&^*P* < 0.05 versus LPS group; ^&&^*P* < 0.01 versus LPS group. **d** The ratio of living cells, apoptosis cells, dead cells and necrotic cells between the LPS group and the LPS + Emo 20 μM group at 4 h was detected. **e** The ratio of living cells, apoptotic cells, dead cells and necrotic cells between the LPS group and the LPS + Emo 20 μM group at 24 h was detected. The data are presented as the mean ± SD. n = 10. *P < 0.05versus apoptosis cell in LPS group; ^#^*P* < 0.05 versus dead cell in LPS group

Granulocytes were isolated and adjusted concentration to 1 × 10^6^/ml. Granulocytes were treated with Emo (5 μM, 10 μM and 20 μM) for 4 h and 24 h. After labeling with Annexin V-FITC and SYTOX, the apoptosis of granulocytes was measured by flow cytometry (Fig. 6a). Our 4 h apoptosis experiment showed that apoptosis of cells did not increase significantly in the LPS group and the LPS + Emo 5 μM group(P>0.05) (Fig. 6b). However, the apoptosis of cells increased significantly in the LPS + Emo 10 μM group and the LPS + Emo 20 μM group, when compared with the control group (*P* < 0.05) (Fig. 6b). We further compared the ratio of living cells, dead cells and necrotic cells between the LPS group and the LPS + Emo 20 μM group, and found that there was no significant difference in the ratio of living cells, dead cells and necrotic cells between the two groups (*P* > 0.05) (Fig. 6d). It suggests that Emo 10 μ M and Emo 20 μ M can promote the apoptosis of granulocytes following LPS stimulation.

The results of the 24 h apoptosis experiment are consistent with those of the 4 h experiment, confirming that 10 μM Emo and 20 μM Emo can promote apoptosis of cells following LPS stimulation at 24 h (*P* < 0.05) (Fig. 6c). Moreover, when compared with the LPS group, dead cells in the LPS + Emo 20 μM group were significantly reduced (*P* < 0.05) (Fig. 6e). It suggests that 20 μM Emo can not only promote the apoptosis of granulocytes following LPS stimulation but also reduce the ratio of dead granulocytes.

### Emo has no protective effect on the rat model of acute lung injury with granulocyte deficiency

On the second day after receiving cyclophosphamide immunosuppression, the rats showed poor mental state, reduced diet and activity, gray and yellow hair, and the above performance was aggravated after injection of LPS.

**Fig. 7:**
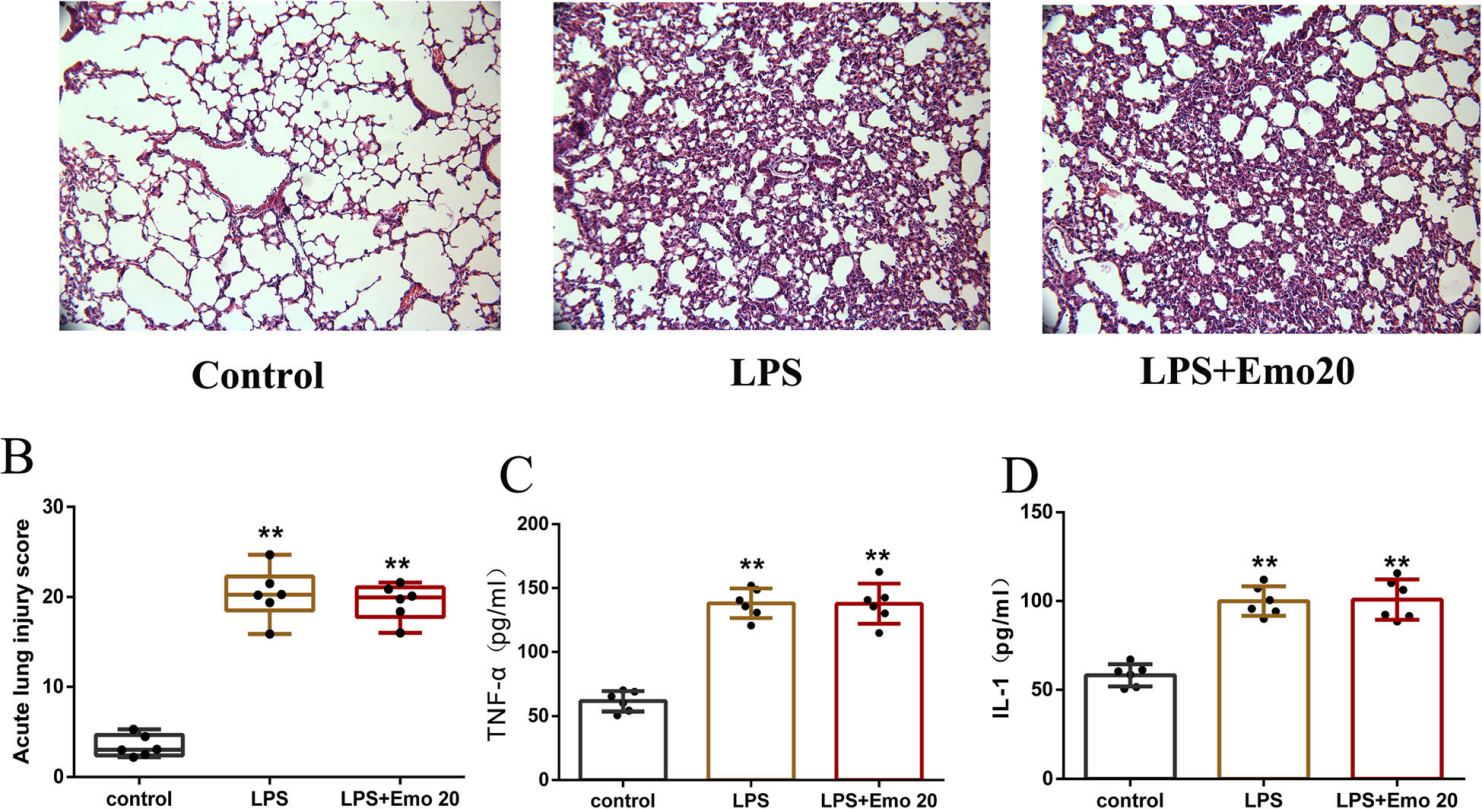
Emo has no protective effect on the rat model of acute lung injury with granulocyte deficiency. **a** The lung tissues were obtained immediately after exsanguination (4 h after LPS), and the effect of Emo 20 mg/kg was assessed histologically in H&E-stained sections (original magnification × 200). **b** Lung injury scores. **c** The lung tissue homogenate TNF-α protein expression and **d** the lung tissue homogenate IL-1β protein expression. The data are presented as the mean ± SD. *n* = 6. **P < 0.01 versus control group

From the H&E staining samples of rat lung tissue, we can see that the lung histology of the control group is normal (Fig. 7a). In LPS group, the lung tissue injury was markedly damaged, with interstitial edema, hemorrhaging, thickening of the alveolar wall, and infiltration of inflammatory cells into the interstitium and alveolar spaces, as evidenced by an increase in lung injury score (*p* < 0.01). In LPS + Emo 20 μM group, the histomorphology of lung tissue was similar to that of LPS, and the injury was severe (Fig. 7b). Emo could not reduce the concentration of TNF- α and IL-1 β in lung tissue homogenate of LPS group. (Fig. 7C-D).

## Discussion

Acute lung injury is a rapid non-cardiogenic bilateral lung infiltration syndrome characterized by alveolar vascular injury, granulocytes (mainly neutrophils) infiltration and accompanied by the release of pro-inflammatory factors [17]. Granulocytes and macrophages play an important role in the process of lung injury, in which granulocytes are the first line of defense against the invasion of pathogens. Macrophages play a key role in the subsequent clearance of apoptotic granulocytes and promoting the regression of inflammation. This study focuses on the effect of Emo on the function of neutrophils in ALI, so as to explore the anti-inflammatory mechanism of Emo.

Granulocytes are the first line of defense against bacterial invasion and make up the largest proportion of leukocytes, which play a vital role in nonspecific immunity [18]. When the body is invaded by foreign microorganisms, granulocytes first gather at the inflammatory lesion site to play a defensive role. Emo (1,3,8-trihydroxy-6- methylanthraquinone) is a naturally occurring anthraquinone derivative and an active ingredient of some Chinese herbs. These herbs have been wildly used as traditional medicines in many countries, especially in eastern Asia. Currently, a number of researchers are focusing on the pharmacological effects of this compound. In the last 5 years, there have been many reports on the anti-tumor and anti-inflammatory effects of Emo [19, 20]. These pharmacological properties suggest that Emo might be a valuable therapeutic option for the prophylaxis and treatment of various diseases, including asthma, atopic dermatitis, osteoarthritis, diabetes and diabetic complications, atherosclerosis, Alzheimer’s disease (AD), hepatic disease and several types of cancers such as pancreatic cancer, breast cancer, hepatocellular carcinoma and lung carcinoma.

In this study, we have identified a unique role for Emo related to the function of granulocytes in LPS-induced inflammatory lung injury. Our data clearly demonstrate that exogenous administration of Emo can reduce the lung injury score of ALI rats induced by LPS. Our experimental results also confirm that treatment with Emo inhibits plasma inflammatory cytokines, such as TNF-α and IL-1β. Decreased plasma TNF-α and IL-1β levels in Emo-treated rats are consistent with the paradigm that Emo reduces the inflammatory response following ALI, in addition to ameliorating the severity of disease [21]. This suggests the potential of Emo as an effective lung protective therapeutic agent.

Neutrophils are over-activated during ALI, which releases neutrophil respiratory burst and ROS to damage the surrounding tissues. Priming of the neutrophil ROS production is believed to be involved in many inflammatory diseases, such as acute respiratory distress syndrome (ARDS), rheumatoid arthritis (RA), atherosclerosis, ischemia-induced tissue injury, hypertension, diabetes, kidney disease, and sepsis [22]. Our experimental results supported this conclusion. In this study, we evaluated the initiation of respiratory burst of suspended granulocytes in vitro using a SOD-inhibited cytochrome c reduction test, and luminol-amplified chemiluminescence to measure ROS [23]. SOD-inhibitable cytochrome c reduction assay is a standard technique for measuring superoxide dismutase production and has the advantages of being a quantitative method that is specific for extracellular superoxide anions [24]. Luminol-amplified chemiluminescence method has the advantages of being very sensitive and able to detect both extracellular and intracellular ROS. We found that Emo reduces granulocytes respiratory burst and ROS production in a dose-dependent manner, thus alleviating the damage from over-active granulocytes to surrounding tissues.

Elastase is a serine protease secreted by polymorphonuclear granulocytes, which plays an important role in many physiological and pathological processes such as the various inflammatory reactions, adult respiratory distress syndrome, and acute lung injury [25]. When exposed to various inflammatory factors, granulocytes release elastase via degranulation, which is then involved in the inflammatory response. Some studies believe that elastase is a destructive elastase that attacks the extracellular matrix and modulates inflammation and tissue remodeling. Its involvement may be direct (tissue damage) or indirect (proinflammatory or proapoptotic) [26–28]. However, it is undeniable that elastase is a necessary condition for neutrophil migration to inflammatory sites, and the proteolytic activity of elastase contributes to the body’s defence against infectious agents by promoting the destruction of pathogenic bacteria [29–31]. Our results show that Emo at the concentration of 10 μM and 20 μM can promote granulocytes elastase release induced by fMLP. To analyze the reasons, we consider that the time point selected in this study is 4 h after the successful establishment of acute lung injury model induced by LPS. This is the peak of inflammation and it is the time when a large number of granulocytes migrate to the inflammatory site. At this time, Emo promotes the release of granulocytes elastase is conducive to granulocyte migration to the site of inflammation to play a bactericidal function.

NETs are a kind of outer network structure, composed of complex three-reticular structure of DNA, and contains the main protein, elastase, myeloperoxidase (MPO), cathepsin G and other antibacterial proteases. It is a special bactericidal mechanism for neutrophils [8]. NETs are a double-edged sword in the inflammation process. Some studies have shown that a large number of extensive NETs formations can damage epithelial cells and endothelial cells, resulting in the spread of inflammation [32]. However, there are still a large number of studies that show that in the acute stages of inflammation, NETs not only capture microorganisms but also limit the diffusion of cytotoxic antimicrobial proteins and reduce host tissue damage [33, 34]. Our research results show that the production of NETs is higher than that of the control group following LPS stimulation of granulocytes, which indicates that the production of NETs is helpful in enabling granulocytes to play a bactericidal function in the acute stages of inflammation. Compared with the LPS group, 5 μM Emo did not increase the generation of NETs, but 10 μM Emo and 20 μM Emo could significantly promote the generation of NETs. This data showed that Emo at concentrations of 10 μM and 20 μM could enhance the bactericidal ability of granulocytes by promoting the production of NETs.

During an inflammatory injury, granulocytes gather at the injury site to exert phagocytosis, an important granulocytes mechanism to kill pathogenic microorganisms. Our study found that Emo promotes phagocytosis of granulocytes: the phagocytosis of S.aureus and E.coli by neutrophils increases with time, reaching a peak at 60 min. At 30 min, 45 min and 60 min, the phagocytosis of S.aureus and E.coli by LPS-stimulated granulocytes was significantly stronger than the control group. We also found that while Emo 5 μM did not promote the phagocytosis of granulocytes, 10 μM Emo and 20 μM Emo significantly promoted granulocyte phagocytosis of S.aureus and E.coli, indicating that Emo needs to reach a certain dose to promote granulocyte phagocytosis.

It is well known that granulocytes have a short life span, mainly due to apoptosis of granulocytes in the circulatory system over time. The resolution of an acute inflammatory response requires subsequent phagocytosis of apoptotic granulocytes by macrophages. Delayed apoptosis of activated granulocytes can lead to persistent acute lung inflammation and can eventually develop into one of the mechanisms of ARDS [35]. In this experiment, we mainly compared the effects of different doses of Emo on LPS-stimulated granulocyte apoptosis. We found that 5 μM Emo had no effect on granulocyte apoptosis after 4 h and 24 h of treatment in the LPS group. However, 10 μM Emo and 20 μM Emo significantly promote the apoptosis of granulocytes in the LPS group after treating the granulocytes for 4 h and 24 h in a dose-dependent manner. This indicates that medium and large doses of Emo can promote the apoptosis of granulocytes and that the larger the dose, the better the effect. We have further compared the effects of 20 μM Emo on dead cells and necrotic cells in the LPS group. The results show that 20 μM Emo can only promote the apoptosis of granulocytes at 4 h, but has no effect on dead cells and necrotic cells. However, at 24 h, 20 μM Emo can not only promote the apoptosis of granulocytes but also reduce the number of dead granulocytes.

Finally, we used cyclophosphamide to inhibit circulating granulocytes in rats, and made a rat model of acute lung injury with granulocyte deficiency. The results showed that Emo could not reduce the pulmonary inflammatory infiltration and lung injury score in the rat model of acute lung injury with granulocyte deficiency, nor could it reduce the levels of inflammatory factors TNF-α and IL-1β in lung homogenate. It is further confirmed that Emo had a protective effect on ALI rats by affecting the function of granulocytes.

In summary, this study demonstrates that Emo alleviates lung injury and reduces the release of inflammatory cytokines in rats with acute lung injury induced by LPS, but has no protective effect on acute lung injury in rats with granulocytes deficiency. Moreover, Emo also downregulated neutrophil respiratory burst and the production of ROS in the LPS-stimulated granulocytes, thus reducing the damage of granulocytes to the surrounding tissues. Emo can also up-regulate the ability of granulocytes to phagocytize bacteria and generate NETs, thereby enhancing the bactericidal ability of granulocytes. In addition, Emo can promote the apoptosis of granulocytes and accelerate the resolution of inflammation. Interestingly, our research has confirmed that small doses of Emo have no obvious effect on granulocyte function. High-dose Emo also had obvious toxic effect on granulocytes. Only Emo (animal: 10–20 mg/kg; cells: 10-20uM) had an obvious effect, and in this range in a dose-dependent manner. Our findings reveal a novel mechanism for Emo to attenuate the inflammatory reaction and shows that Emo could be exploited therapeutically for acute lung injury.

## Conclusion

Emo has a protection effect on LPS-induced acute lung injury rats. It can alleviate lung injury and reduces the release of inflammatory cytokines, by affecting the various function of granulocytes. The appropriate dose (animal: 10–20 mg/kg; cells: 10-20 μM) has the best effect.

## Materials and methods

### Reagents

LPS (*Escherichia coli* serotype 055:B5), formyl methionyl leucyl phenylalanine (fMLP), interleukin-8 (IL-8), phorbol ester (PMA), cell chromatography C (Cytochrome C), superoxide dismutase (SOD), Elastase, Hydroxyethylpiperazine Ethylsulfonic Acid (HEPES) and Emo were obtained from Sigma-Aldrich (St Louis, MO, USA). Tumor necrosis factor-α (TNF-α) and interleukin-1β (IL-1β) ELISA kits were obtained from R&D Systems (Minneapolis, MN). Anti-Myeloperoxidase (MPO) antibody (abcam, ab65871), MNase, RP-1 antibody (BD 550002), SYTOX Green, and Annexin V-FITC were obtained from eBioscience (San Diego, CA). pHrodo red *E. coli* (Cat.No.4615), pHrodo green S.aureus (Cat. No. 4620) were obtained from Sartorius (Göttingen, Germany). RPMI 1640, fetal bovine serum (FBS), trypsin, and enzyme-free cell dissociation buffer were purchased from Gibco (Grand Island, NY, USA). Penicillin and streptomycin in saline citrate buffer were from Invitrogen (Carlsbad, CA, USA). Other chemical reagents are of analytical grade.

### Animals, experimental procedure, and treatments

Experiments were performed on adult male Sprague Dawley rats (250–300 g; Shanghai Experimental Animal Center of China). Rats were provided with water and food ad libitum. The use of animals in this study was approved by Animal Studies Ethics Committee of the Second Affiliated Hospital of Wenzhou Medical University.

Rats were randomized into five groups (*n* = 6): control group, LPS group, LPS + Emo 5 mg/kg group, LPS + Emo10 mg/kg group and LPS + Emo 20 mg/kg group. In LPS group, rats received LPS (20 mg/kg) through the tail vein. LPS was dissolved in 0.9% normal saline. In the Emo group, rats received Emo (5 mg/kg, 10 mg/kg and 20 mg/kg) via intraperitoneal injection 30 min before LPS exposure. Emo was dissolved in 100% DMSO at a concentration of 200 mg/mL and diluted in saline to the final concentration of 1 mg/ml. The action time of LPS-induced acute lung injury in rats was 4 h.

### Pathological studies

Rats were anesthetized with chloral hydrate (7 ml/kg, intraperitoneally), intubated and connected to the animal ventilator (respiratory parameters are tidal volume: 10 ml / kg and respiratory rate: 40-60 bpm) 4 h after injection of LPS. After anesthesia and mechanical ventilation with pure oxygen for 1 h, rats were killed by cutting off the abdominal aorta and bloodletting. Rats were subjected to thoracotomy and PBS (25 ml/min) was injected into the right ventricle to flush the pulmonary vessels. Finally, the right lower lung lobe of rats was cut and fixed in 4% paraformaldehyde for 24 h at room temperature, and 4 μ m sections were embedded in paraffin and stained with hematoxylin and eosin (H&E) for light microscopy analysis. The rest of the lung tissue was frozen in liquid nitrogen for 48 h and stored in refrigerator at-80 °C.

A semi-quantitative scoring system was adopted to evaluate lung injury, which included alveolar congestion, alveolar hemorrhaging, neutrophil infiltration or aggregation in the airspace or vessel wall, and alveolar wall/hyaline membrane thickness and inflammatory cell infiltration. The grading scale for the light microscopy pathologic findings was as follows: 0 = no injury; 1 = slight injury (25%); 2 = moderate injury (50%); 3 = severe injury (75%); and 4 = very severe injury (almost 100%). The results were graded from 0 to 4 for each item, as described previously. The four variables were summed to represent the lung injury score (total score: 0–16).

### Determination of inflammatory cytokines in lung homogenate by enzyme-linked immunosorbent assay (ELISA)

Part of the right lung from individual rats was homogenized and centrifuged, and the levels of TNF-α and IL-1β in the resulting tissue supernatants were determined using TNF-α and IL-1β ELISA kits.

After ultrasonic lysis of lung homogenate, the supernatant was obtained by centrifugation at 4 °C for 5000 r/min for 15 min. Follow the reagent instructions. 100 μ l of standard or sample to be tested was added to each hole, and the reaction plate was fully mixed and placed at 37 °C for 30 min. Wash the reaction plate fully with washing solution for 4 times and print it on the filter paper for 6 times. 100 μ l of enzyme-labeled antibody working solution was added to each well. Put the reaction plate at 37 °C for 30 min. The washing board is the same as before. 100 μ l of substrate working solution was added to each hole and reacted in the dark at 37 °C for 15 min. Add 100 μ l terminating liquid to each hole and mix well. The absorbance value of 450 nm was measured by enzyme labeling instrument within 30 min.

### Separation and of rat granulocytes

20 ml of heparinized fresh rat blood was treated with dextran to induce sedimentation of the red blood cells. Prepare a Percol gradient in a 15 ml Falcon Tube by first pipetting 5 ml 56% Percol, then put the sucker to the bottom of the tube, and slowly pipetting 2.5 ml 80% Percol to the bottom. Then carefully draw up the plasma and white blood cell suspension from the blood sample with a pipette and slowly layer them on top of the Percol gradient.4 °C, 220 g, centrifuge for 20 min, accelerate to 1, decelerate to 0, remove the top layer of serum, suck out the granulocyte layer, add PBS to wash twice, the cells were resuscitated with RPMI-1640 medium containing 5% FBS and then counted so that the cell concentration was 1 × 10^6^/ml.

Granulocytes were divided into five groups: control group, LPS group (100 ng/ml), LPS+ Emo 5 μM group, LPS+ Emo 10 μM group and LPS+ Emo 20 μM group. Stimulation with Emo was performed for 30 min prior to LPS treatment.

### Immunofluorescence staining

Granulocytes were isolated and adjusted concentration to 5 × 10^5^/ml. They were centrifuged for 5 min in order to fix the cells on glass slides and dried. Granulocytes were fixed with 4% paraformaldehyde at room temperature for 15 min. Then they were permeabilize with 0.5%TritonX-100PBS for 20 min and blocked with blocking solution buffer for 30 min. Granulocytes were incubated overnight at 4 °C with primary antibody (MPO antibody 1: 500), and then incubated the secondary antibody (1:200) onto glass slides for 1 h at room temperature in moist environmental box. DAPI was diluted into PBS in accordance with the instructions and added onto glass slides for 5 min at room temperature. 20 μL of mounting medium was promptly added onto the slide, then put a cover slip on it, followed by mounting the coverslip with nail enamel. Finally, the slides were observed under a fluorescence microscope.

### Cell counting kit-8 (CCK8 assay)

Granulocytes were isolated and adjusted concentration to 1 × 10^6^/ml. Granulocytes were added to 96-well plate (100 μ l per hole). Three multiple holes and blank control holes were set up at the same time (no cells). Emo was added into test wells as an inhibitor, which was divided into five concentration gradients of 5 μ M, 10 μ M, 20 μ M, 40 μ M and 80 μ M. PBS was used as negative control. After 4 h of Emo intervention, 10 μ l of CCK-8 reagent was added into each well. The plate was cultured in 5%CO_2_ incubator at 37 °C for 3 h, the OD value of each well of wavelength 450 nm was detected by enzyme labeling instrument. Cell inhibition rate (IC) can be calculated according to the formula:

$$ \mathrm{Cell}\ \mathrm{inhibition}\ \mathrm{rate}\ \left(\mathrm{IC}\right)=\left[\left(\mathrm{control}\ \mathrm{group}\ \mathrm{OD}\ \mathrm{value}-\mathrm{experimental}\ \mathrm{group}\ \mathrm{OD}\ \mathrm{value}\right)/\left(\mathrm{control}\ \mathrm{group}\ \mathrm{OD}\ \mathrm{value}-\mathrm{zeroing}\ \mathrm{group}\ \mathrm{OD}\ \mathrm{value}\right)\times \right]\ 100\% $$

### Respiratory burst detection

The reactive oxygen species released by the activated inflammatory cells can reduce the membrane non-penetrating cytochrome C. The reduced cytochrome C has an absorption peak at 550 nm. Therefore, the amount of reduced cytochrome C is measured using a spectrophotometer. The amount of active oxygen produced can be inferred from this data. ①Set control group, LPS group and 3 Emo groups, and the appropriate amount of Emo was added in each group, 100 μ l of cytochrome C (1.5 mg/ml) and 100 μl neutrophils (2 × 10^7^/ml) was then added; ②10 μl SOD (5000 U/ml) was added, and the corresponding dose was added to the test group, equilibrated in a 5% CO_2_ incubator at 37 °C for 10 min; ③10 μ l cytochalasin B (1 mmol/L) was added to each group and after 3 min, 10 μ l fMLP (0.1 mmol/L) was added for a total of 1 ml and each group was incubated in a 5% CO_2_ incubator at 37 °C for 30 min; ④ Each group was removed and centrifuged at 2000 r/min for 10 min;⑤ Supernatant was collected and the OD value was measured with a spectrophotometer. Since the production of O_2_^−^ and the decrease in cytochrome C is in a 1:1 mol stoichiometric relationship, the yield of O_2_^−^ is easily calculated. The millimolar extinction coefficient of the 1 cm optical path is 21.1, and the amount of O_2_^−^ produced by 2 × 10^6^ / ml of cells in 1 ml of the solution with a diameter of 1 cm can be directly calculated according to the formula:

$$ \mathrm{OD}\times 47.4=\mathrm{nmol}\ {\mathrm{O}}_{\overline{2}}/2\times 106\mathrm{cells}/\mathrm{time}\ \mathrm{unit}\ \mathrm{test}\ \mathrm{group}\ {\mathrm{O}}_{\overline{2}}\ \mathrm{inhibition}\ \mathrm{rate}=\left(\mathrm{control}\ {\mathrm{O}}_{\overline{2}}\ \mathrm{content}-\mathrm{test}\ \mathrm{group}\ {\mathrm{O}}_{\overline{2}}\ \mathrm{content}\right)/\mathrm{control}\ \mathrm{group}\ {\mathrm{O}}_{\overline{2}}\ \mathrm{content}\times 100\%. $$

### Elastase release assay

The detection of granulocyte elastase release was mainly carried out by Elastase Activity Assay kit (ab204730). Five elastase solution test groups were prepared: Control group, LPS group, and Emo group (reaction solution concentration: 5 μ M, 10 μ M, 20 μ M). ①The isolated granulocytes were rinsed twice with PBS (pH 7.4), the number of cells was adjusted to 1 × 10^7^/ml, 500 μl of solution was added to each group, and the corresponding drugs were added. Finally, each group was supplemented with PBS to 600 μl. The groups were pre-incubated for 30 min at 37 °C in a 5% CO 2 incubator. In addition to Control group, 6 μl of cytochalasin B (1 mmol/L) and LPS (100 ng/ml) was added to each group and cultured at 37 °C in a 5% CO _2_ incubator for 20 min. The tubes were then placed in an ice-water bath to terminate the reaction. They were centrifuged at 1500 r/min for 5 min, and the supernatant was dispensed and stored at − 80 °C until use. ②Elastase determination. Elastase standard was diluted with PBS: 50, 37.5, 25, 18.75, 12.5, 9.38, 6.25, 4.69, 3.125 series concentrations (μ g /ml) and a PBS blank were used to generate a standard curve. Using a 96-well microtiter plate containing a standard or a 50 μ l sample to be tested, 100 μ l buffer was added (containing elastase substrate 1 mmol/L, HEPES 0.1 mol/L, NaCl 0.5 mol/L, pH 7.5). The OD value was read at 405 nm by a microplate reader (the emodin absorption is at 405 nm), and then cultured at 37 °C in a 5% CO _2_ incubator for 60 min. The OD value at 405 nm was then read again, and the difference between the two OD values was recorded. The OD value of the substrate decomposes, and the elastase content is calculated according to the standard curve.

### Measuring NETs production

Clear 96-well flat-bottomed plates were prepared, and 100 μl of granulocytes were added to the relevant wells. Lipopolysaccharide (LPS, 100 ng/ml), interleukin-8 (IL-8, 100 ng/ml), phorbol ester (PMA, 1.5 ng/ml) and N-formylthionyl-leucyl-phenylalanine (fMLP, 1000 ng/ml) were used to treat the cells respectively. The control group was treated with an equal volume of medium. They were incubated for 3 h at 37 °C in a 5% CO_2_ incubator. SYTOX Green was diluted 1:500 (5 mM Stock; 1ul SYTOX Green into 499ul PBS), and then stored in the dark. 20 μl of diluted SYTOX green was added to each well using a fresh tip for each well. 1 μl of MNase was added to each well using a fresh tip for each well. They were then incubated at room temp for 10 min in the dark. Samples were transferred to 0.5 ml micro-centrifuge tubes without any pipetting of the liquid up and down. They were immediately centrifuged at 5000 rpm for 10 min in the micro-centrifuge before 160 μl of the supernatant was removed and transferred to a black 96-well flat-bottomed plate. Fluorescence was measured immediately (programme: Gen5; excitation 485 nm, emission 528 nm with optics position in top 50% of well with a 10-s ‘medium’ shake immediately prior to read).

### Measuring ROS production by isolated granulocytes

Following isolation, cells were resuspended at 1 × 106/ml in HBSS (with Ca^2+^ and Mg^2+^) (4.5 ml total) in 15 ml Falcon. 100 μl of granulocytes were added to each well of a 96-well plate. Cells were stimulated with Luminol (0.5 mM), IL-8(1.25 nM), fMLP (2.5 μM), and PMA (25 nM) for 1 h. The luminometer was set up and the ROS level was tested on the instrument.

### Measuring the phagocytosis of granulocytes

Granulocytes were isolated and adjusted concentration to 1 × 10^6^/ml. Following LPS and Emo treatment, granulocytes were inoculated into 96-well plates at 100 μl/well. pHrodo red *E. coli* and pHrodo green *S. aureus* were added to granulocytes respectively to stimulate granulocytes for 30 min, 45 min, and 60 min. Granulocytes were incubated at 37 °C in a 5% CO_2_ incubator in the dark, and then centrifuged at 250 g and 4 °C for 5 min to remove the supernatant. The cells were resuspended with 100ul of 2% PBS/BSA, and this was repeated twice before the cell suspension from each well was transferred into flow tubes. 100 μl of 2% PBS/BSA was added to each tube, gently mixed and placed on ice. Finally, the phagocytosis of granulocytes was measured using flow cytometry.

### Measuring the rate of apoptotic granulocytes

Granulocytes were isolated and inoculated into six-well plates at an adjusted concentration of 1 × 10^6^/ml. The groups were divided into groups and treated for 4 h and 24 h. Cells were harvested as normal and cells were transferred to the appropriate FACS tubes. They were centrifuged at 600 g for 4 min before the supernatant was poured off. Cells were resuspended in 200 μl Annexin V buffer to wash the cells and then pelleted again. The cells were incubated in 100 μl Annexin V-FITC diluted 1:100 in Annexin V buffer for 15–20 min on ice and protected from the light. 200 μl Annexin V buffer was added to each tube. SYTOX was removed from the freezer and defrosted while being protected from the light. A SYTOX stock diluted 1:500 in Annexin V buffer was prepared. Immediately prior to running the sample on the CyAN, 30 μl of the SYTOX solution was added to each tube and they were vortexed well to mix. The FITC and Violet 1 channels on the FACS machine were used to measure.

### To establish a rat model of acute lung injury with granulocyte deficiency

Cyclophosphamide (CTX) is a kind of non-specific chemotherapeutic drug in cell cycle, which is widely used in clinic. It can kill the cells in each phase of the proliferation cycle and inhibit the number of leukocytes in bone marrow.

In this experiment, rats were injected intraperitoneally with cyclophosphamide (75 mg /kg)4 days before and 1 day before the acute lung injury induced by LPS. And 1 day before the acute lung injury model was prepared and 1 day after the model was prepared, the number of granulocytes in rat tail vein blood was less than 2 × 10^5^/ml by using the blood cell count version technology, Therefore, the rat model of acute lung injury with granulocytedeficiency was successfully prepared.

### Statistical analysis

The data represent the mean ± SD. There were no missing, lost, or excluded data. Based on previous experience, no prior power analysis was conducted; all data were analyzed by one-way ANOVA followed by Tukey’s post-hoc test for multiple comparisons. All tests were two-sided, and significance was determined at the *p* < 0.05 level. Statistical analyses were performed using Prism 6.0 software (GraphPad Software, San Diego, CA).

## Data Availability

All data generated or analyzed during this study are included in this published article and are available from the corresponding author upon request.

## Abbreviations

ALI: Acute lung injury
ARDS: acute respiratory distress syndrome
Emo: Emodin
LPS: lipopolysaccharide
TNF-α: tumor necrosis factor-α
IL-1β: Interleukin-1β
NETs: neutrophil extracellular traps
ROS: reactive oxygen species
fMLP: formyl methionyl leucyl phenylalanine
IL-8: interleukin-8
PMA: phorbol ester
Cytochrome C: cell chromatography C
SOD: superoxide dismutase
HEPES: Hydroxyethylpiperazine Ethylsulfonic Acid
FBS: fetal bovine serum
ELISA: enzyme-linked immunosorbent assay
CCK8: Cell Counting Kit-8
CTX: Cyclophosphamide
MPO: myeloperoxidase
RA: rheumatoid arthritis
